# Toll-like receptor linked cytokine profiles in cerebrospinal fluid discriminate neurological infection from sterile inflammation

**DOI:** 10.1093/braincomms/fcaa218

**Published:** 2020-12-17

**Authors:** Simone M Cuff, Joseph P Merola, Jason P Twohig, Matthias Eberl, William P Gray

**Affiliations:** Division of Infection and Immunity, School of Medicine, Cardiff University, Cardiff, UK; Systems Immunity Research Institute, Cardiff University, Cardiff, UK; Division of Psychological Medicine and Clinical Neurosciences, School of Medicine, Cardiff University, Cardiff, UK; Neurosciences and Mental Health Research Institute (NMHRI), Cardiff University, Cardiff, UK; BRAIN Biomedical Research Unit, Cardiff University, Cardiff, UK; Department of Neurosurgery, University Hospital Wales, Cardiff, UK; Division of Infection and Immunity, School of Medicine, Cardiff University, Cardiff, UK; Systems Immunity Research Institute, Cardiff University, Cardiff, UK; Division of Infection and Immunity, School of Medicine, Cardiff University, Cardiff, UK; Systems Immunity Research Institute, Cardiff University, Cardiff, UK; Division of Psychological Medicine and Clinical Neurosciences, School of Medicine, Cardiff University, Cardiff, UK; Neurosciences and Mental Health Research Institute (NMHRI), Cardiff University, Cardiff, UK; BRAIN Biomedical Research Unit, Cardiff University, Cardiff, UK; Department of Neurosurgery, University Hospital Wales, Cardiff, UK

**Keywords:** central nervous system infections, external ventricular drain, ventriculoperitoneal shunt, cytokines, pathway analysis

## Abstract

Rapid determination of an infective aetiology causing neurological inflammation in the cerebrospinal fluid can be challenging in clinical practice. Post-surgical nosocomial infection is difficult to diagnose accurately, as it occurs on a background of altered cerebrospinal fluid composition due to the underlying pathologies and surgical procedures involved. There is additional diagnostic difficulty after external ventricular drain or ventriculoperitoneal shunt surgery, as infection is often caused by pathogens growing as biofilms, which may fail to elicit a significant inflammatory response and are challenging to identify by microbiological culture. Despite much research effort, a single sensitive and specific cerebrospinal fluid biomarker has yet to be defined which reliably distinguishes infective from non-infective inflammation. As a result, many patients with suspected infection are treated empirically with broad-spectrum antibiotics in the absence of definitive diagnostic criteria. To begin to address these issues, we examined cerebrospinal fluid taken at the point of clinical equipoise to diagnose cerebrospinal fluid infection in 14 consecutive neurosurgical patients showing signs of inflammatory complications. Using the guidelines of the Infectious Diseases Society of America, six cases were subsequently characterized as infected and eight as sterile inflammation. Twenty-four contemporaneous patients with idiopathic intracranial hypertension or normal pressure hydrocephalus were included as non-inflamed controls. We measured 182 immune and neurological biomarkers in each sample and used pathway analysis to elucidate the biological underpinnings of any biomarker changes. Increased levels of the inflammatory cytokine interleukin-6 and interleukin-6-related mediators such as oncostatin M were excellent indicators of inflammation. However, interleukin-6 levels alone could not distinguish between bacterially infected and uninfected patients. Within the patient cohort with neurological inflammation, a pattern of raised interleukin-17, interleukin-12p40/p70 and interleukin-23 levels delineated nosocomial bacteriological infection from background neuroinflammation. Pathway analysis showed that the observed immune signatures could be explained through a common generic inflammatory response marked by interleukin-6 in both nosocomial and non-infectious inflammation, overlaid with a toll-like receptor-associated and bacterial peptidoglycan-triggered interleukin-17 pathway response that occurred exclusively during infection. This is the first demonstration of a pathway dependent cerebrospinal fluid biomarker differentiation distinguishing nosocomial infection from background neuroinflammation. It is especially relevant to the commonly encountered pathologies in clinical practice, such as subarachnoid haemorrhage and post-cranial neurosurgery. While requiring confirmation in a larger cohort, the current data indicate the potential utility of cerebrospinal fluid biomarker strategies to identify differential initiation of a common downstream interleukin-6 pathway to diagnose nosocomial infection in this challenging clinical cohort.

## Teaser

Rapid determination of nosocomial infection causing neurological inflammation in the CSF is a significant unmet need in clinical practice. Using pathway analysis in samples from neurosurgical patients with diagnostic equipoise, Cuff *et al.* report that elevated IL-17 and IL-23 levels consistent with bacterial wall peptidoglycan activation of Toll-like-receptors, identifies nosocomial infection.

## Graphical Abstract

**Graphical Abstract:**
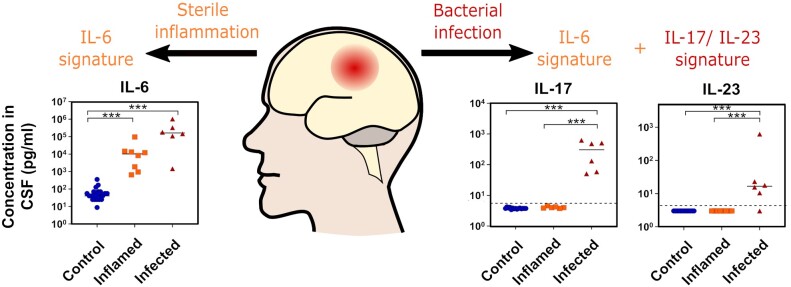

## Introduction

Neurological infection continues to pose a significant burden of disease and is responsible for an estimated 20.4 million disability-adjusted life years globally (95% confidence interval (CI) 17.4–23.4 million (Feigin *et al.*, 2019). Nosocomial neurological infection differs significantly from community-acquired infection, as it usually develops after neurosurgical or neurological interventions, has a distinct bacteriological profile and by definition occurs against a background neurological illness. These all conspire to make early and accurate differential diagnosis difficult but mandatory for the optimal management of these often critically ill patients (van de Beek *et al.*, 2010). Moreover, many acute neurological and neurosurgical conditions have a prominent inflammatory component from central nervous system (CNS) damage, and it is especially challenging to diagnose superimposed nosocomial infection against a background of an already activated innate immune response (Tunkel *et al.*, 2017). The lack of accurate diagnostic tests at the point of clinical equipoise is a critically important unmet need with significant clinical and healthcare cost implications (Beer *et al.*, 2008; Dorresteijn *et al.*, 2019; Mallucci *et al.*, 2019; Sorinola *et al.*, 2019; Zervos and Walters, 2019).

Recent cranial neurosurgery is a major risk factor for nosocomial ventriculitis or meningitis (van de Beek *et al.*, 2010). The risk varies significantly with surgical procedure, duration and whether devices are implanted (Kourbeti *et al.*, 2015). The most common implanted devices are either temporary external ventricular drains (EVD) or permanent ventriculoperitoneal (VP) shunts for controlling hydrocephalus (Fernández-Méndez *et al.*, 2019). Both EVD and VP shunt insertion carry a significant risk of infection, with recent large UK prospective studies showing rates of 9.3% in 495 EVD insertions (Jamjoom *et al.*, 2018) and 6% in 1594 first VP shunts (Mallucci *et al.*, 2019).

Nosocomial infection introduced by EVDs and VP shunt surgery is particularly challenging to diagnose against a background of significant neurological disease and/or inflammation caused by recent surgery. The conventional clinical symptoms and signs of community-acquired neurological infection are unreliable, with the classic triad of fever/nuchal rigidity/altered mental status or headache being only partially or fully present in 50–95% of patients (van de Beek *et al.*, 2010; Viallon *et al.*, 2016). Gram staining of CSF although rapid and highly specific, has poor sensitivity for neurosurgical infections with only 20% of healthcare-associated ventriculitis and meningitis showing a positive CSF Gram stain (Srihawan *et al.*, 2016). While CSF culture remains the mainstay of diagnosis, both EVD and VP shunt infections have similar bacteriological profiles, largely *Staphylococcus aureus* and coagulase-negative *Staphylococcus*, which typically cause a low-grade inflammation from within a biofilm (Gutierrez-Murgas and Snowden, 2014), often making bacteriological detection from CSF difficult. Not only does bacterial culture have a high false-negative rate (up to one third in some cases) (Wu *et al.*, 2013), it usually requires 24–48 h to generate definitive results, frequently necessitating blind empirical interim antibiotic treatment. Additionally, microbiological culture often yields false positives due to contamination during sampling or fails to result in the growth of any organism in partially responsive empirically pre-treated cases. The addition of biochemical measures such as CSF glucose and protein levels, C-reactive protein and procalcitonin to microbiological and clinical factors have limited diagnostic value for diagnosing nosocomial infection, because of the often low-grade nature of the infecting pathogens resulting in smaller bacteriological metabolic perturbations from normal (for reviews see Dorresteijn *et al.*, 2019; Zervos and Walters, 2019).

Promising results in the diagnosis of community-acquired bacterial meningitis (Prasad *et al.*, 2014; Srinivasan *et al.*, 2016) have generated considerable interest in investigating a variety of inflammatory biomarkers to diagnose nosocomial CSF infection. However, many of these studies are based on retrospective case series and do not always distinguish between CSF samples taken at initial clinical diagnostic equipoise and later samples collected after empirical or culture-positive treatments have commenced. Many are diagnosed *posthoc* and although automated group analysis is accurate for group prediction (van Mourik *et al.*, 2015), its positive predictive value remains poor (van Mourik *et al.*, 2012). Moreover, most studies investigate single or small numbers of CSF factors, with limited combinatorial analyses confounding the investigation of biologically plausible underlying signalling mechanisms. A single study (Skar *et al.*, 2019*a*) in a rodent EVD model showed a trend towards a promising pattern of interleukin (IL)-10, IL-1β, chemokine ligand 2 (CCL2) and CCL3, which differentiated infection from inflammation.

In this prospective study, we examined a battery of 182 inflammatory and neurological markers in CSF obtained from EVDs, shunts or via lumbar puncture at the point of clinical diagnostic equipoise, across a spectrum of neurosurgical patients, to determine whether CSF biomarkers can distinguish between inflammatory processes due to infective and non-infective causes. Uniquely, we then used a modified pathway analysis method to examine the biological underpinnings of the biomarker changes identified. We found that increased levels of inflammatory cytokines related to IL-6 were excellent indicators of inflammation, as was described previously, but that they could not distinguish between bacterially infected and uninfected patients. By performing a two-tiered statistical analysis, we show a potential biomarker signature of diagnostic relevance, consisting of raised IL-6 or oncostatin M (OSM) levels in the CSF for detecting general CNS inflammation, and additionally raised IL-17 together with raised IL-12p40 or IL-23 levels as indicative of a bacterial cause for the inflammation. Pathway analysis identified toll-like receptor (TLR) activation as the likely underlying signalling pathway discriminating infection from a common background of IL-6 pathway mediated neuroinflammation.

## Materials and methods

### Sample population

Between March 2017 and March 2018, 38 patients in whom CSF sampling was clinically indicated were consented for additional donation and sampling of CSF and serum for the purpose of this study. CSF samples were obtained from the Wales Neurosciences Research Tissue Bank (WNTRB Ethics Rec Ref: WA/19/0058; Requisition No. 019). CSF samples were collected by trained individuals as part of routine clinical care, transferred on ice and centrifuged (4500 rpm, 10 min at 4°C) to remove cells and debris within 30 min of collection. The acellular supernatant was then aliquoted (300 µl) and frozen at −80°C for storage in the WNTRB until analysis.

Patients were categorized into three groups that were mixed with respect to the underlying neurological condition (Table 1). Index patients were prospectively identified and categorized on the basis of clinical diagnostic equipoise for nosocomial infection, and all underwent CSF sampling (Supplementary Table 1). This sampling at the point of equipoise was intentional, as this is the clinical timepoint at which an investigation of biomarkers for diagnostic utility is the most clinically relevant. This group was later characterized into two diagnostic subgroups. Nosocomial infection (‘Infected group’) was diagnosed on the basis of positive CSF Gram-stain or bacteriological culture that was not considered a contaminant, or pragmatically in cases that were Gram-stain negative, on the basis of CSF pleocytosis, elevated serum inflammatory markers and clinical signs including fever, meningism and altered consciousness [Infectious Diseases Society of America (IDSA) guidelines (Tunkel *et al.*, 2017) and as previously reported (Lozier *et al.*, 2002; Keong *et al.*, 2012; Jamjoom *et al.*, 2018). The ‘Inflamed group’ (chemical meningitis or subarachnoid haemorrhage) had a clinically evident inflammatory condition in whom infection was not diagnosed, and the ‘Control group’ were patients with normal pressure hydrocephalus or idiopathic intracranial hypertension undergoing CSF sampling for management and who had no evidence of infection or inflammation. For patients in whom intra-patient variation was measured, subsequent samples during the infectious or inflammatory episode and subsequent recovery were used and analysed separately.

**Table 1:** Patient characteristics

	Control (*n* = 24)	Inflamed (*n* = 8)	Infected (*n* = 6)
	Mean ± SD (range)	Mean ± SD (range)	Mean ± SD (range)
**M/F**	2/22	3/5	4/2
**Age**	43.5 ± 19.5 (21–95)	42.9 ± 15.1 (28–67)	43.7 ± 8.9 (31–60)
**Neurological diagnosis**	19 IIH 5 NPH	4 SAH 4 Post-surgical chemical meningitis Trigeminal neuralgia Epidermoid cyst Chiari malformation (×2)	1 Pituitary adenoma 1 Colloid cyst 2 Haemangioblastoma 1 SAH 1 IIH
**Indication for CSF sampling**	15 symptom control 9 shunt malfunction	4 SAH-induced hydrocephalus 4 Post-surgical chemical meningitis	3 meningitis 1 meningitis/ventriculitis 1 shunt infection 1 infected pseudomeningocele
**Method of CSF sampling**	14 LP 3 LD 3 VP shunt 1 shunt tap 3 shunt revision	1 EVD (SAH) 7 LP	2 LP 2 LD 1 EVD 1 shunt revision
**SERUM**			
**Haemoglobin**	134 ± 27.66 (113–166)	126.4 ± 10.3 (111–139)	129.7 ± 14.6 (108–146)
**Red blood cells**	4.86 ± 2.80 (3.83–9.5)	4.06 ± 0.43 (3.4–4.6)	4.39 ± 0.57 (3.52–5.08)
**White blood cells**	8.05 ± 3.92 (5.9–12.6)	9.4 ± 1.53 (5.6–11)	13.22 ± 5.00 (5.7–22.3)
**Neutrophils**	5.19 ± 3.17 (3.0–9.1)	6.6 ± 2.07 (2.1–9.1)	10.1 ± 4.87 (3–19.2)
**Lymphocytes**	1.92 ± 1.21 (0.7–3.2)	2.05 ± 1.17 (0.5–4.4)	2.01 ± 0.91 (0.7–3.7)
**CSF**			
**Red cell count** **White cell count** **% Polymorph count** **Gram stain** **Culture**	1762.9 ± 9103.6 (0–17 558) 3.83 ± 19.6 (0–42) n/a No organisms seen No growth on extended cultures	47 547.5 ± 110 639.4 (26–339 616) 77.13 ± 118.6 (0–384) 17.5 ± 23.58 (5–72) No organisms seen No growth on extended culture	134 ± 150.7 (1–395) 1527 ± 2261.3 (11–6480) 89 ± 5.79 (81–95) 2× Gram positive cocci 2× coagulase-negative *S. aureus* ^*^ 4 clinically diagnosed bacterial infection

Geometric means and SD used for CSF RBC counts. RBC counts were significantly higher in the CSF of inflamed compared to infected patients. CSF white cell counts were significantly raised in the inflamed group and the infected group compared to control patients by Kruskal–Wallis test with Dunn’s correction for multiple comparisons. Adjusted *P*-values were used.

* *P *<* *0.05,

** *P *<* *0.01.

Other measured parameters were not significantly different between groups.

EVD = external ventricular drain; IIH = idiopathic intracranial hypertension; LD = lumbar drain; LP = lumbar puncture; NPH = normal pressure hydrocephalus; SAH = subarachnoid haemorrhage; VP = ventriculoperitoneal.

Linked-anonymized clinical data were collected and stored for subsequent analysis. This included patient demographics, past medical history, concurrent medication particularly if immunosuppressant, diagnoses, use of antibiotics, method of sampling, indication for sampling, serum and CSF cell counts (Hb, white cell counts, red blood cell (RBC) counts), and the results of the extended microbiological culture.

### Screening of CSF biomarkers

One hundred and eighty-two biomarkers were measured in 1 μl CSF samples by proximity extension assay by the Olink Analysis Service at Olink laboratories (Olink Proteomics AB, Uppsala, Sweden) using pairs of target-specific antibodies with unique barcoded DNA oligonucleotides contained in the Inflammation I and Neurology panels (Supplementary Table 2), as per the manufacturer’s instructions. Data were normalized to standard plasma controls and are expressed as normalized protein expression. Normalized protein expression is a semi-quantitative log_2_ unit which reflects protein concentration but does not quantify it. Therefore, it is suitable for comparisons between samples but is not a definitive quantitation in itself.

**Table 2:** Potential biomarkers for differentiation between non-inflamed patients, non-infectious inflammation and infection

**Control** versus **all inflamed/infected patients**				**Inflamed** versus **infected patients**	
Biomarker	AUC	Biomarker	AUC	Biomarker	AUC
IL-6	1.000	EN-RAGE	0.946	IL-17	0.979
OSM	1.000	CCL8	0.946	IL-12p40	0.958
LIF	1.000	CXCL10	0.944	IL-12p70	0.938
CXCL8	0.981	CCL23	0.936	CCL3	0.938
G-CSF	0.978	IL-10	0.936		
CCL7	0.975	CXCL6	0.929		
CXCL1	0.974	CD6	0.920		
MMP-1	0.971	CASP8	0.918		
CCL20	0.967	CCL3	0.916		
MMP-10	0.963	CXCL5	0.916		
CCL4	0.961	CCL13	0.915		
CCL2	0.947	CXCL11	0.910		

Biomarkers showing significant differences between non-inflamed and inflamed brain, and inflammation due to non-infective and infective causes and corresponding area under the curve (AUC) for the precision-recall curve.

**Table 3:** Key to Fig. 5.

A	IL-17 signalling in fibroblasts
B	Role of IL-17 in psoriasis
C	IL-17 signalling in airway cells
D	IL-17 signalling
E	Role of IL-17 in arthritis
F	Role of IL-17F in allergic inflammatory airway diseases
G	IL-71 signalling in gastric cells
H	Differential regulation of cytokine production in macrophages and T helper cells by IL-17 & IL-17F
I	Th17 activation pathway
J	Differential regulation of cytokine production in intestinal epithelial cells by IL-17 and IL-17F
K	Role of hypercytokinaemia/hyperchemokinaemia in the pathogenesis of influenza
L	Chemokine signalling
M	Agranulocyte adhesion and diapedesis
N	Granulocyte adhesion and diapedesis
O	TREM1 signalling
P	Toll-like receptor signalling
Q	Activation of IRF by cytosolic pattern recognition receptors
R	Pathogenesis of multiple sclerosis
S	Type I diabetes mellitis signalling
T	Adrenomedullin signalling pathway
U	Systemic lupus erythematosus in T cell signalling pathway
V	Dendritic cell maturation
W	Acute phase response signalling
X	LXR/RXR activation
Y	MSP/RON signalling pathway
Z	Osteoarthritis pathway
AA	IL-10 signalling
AB	IL-6 signalling

Quantitation of inflammatory mediators was independently performed in our laboratory with either the Luminex platform using the manufacturer’s instructions (CXCL5, CXCL8, CXCL10, extracellular newly identified receptor for advanced glycation end-products binding protein (EN-RAGE/S100A12), IL-6, IL-12p40, IL-17, LIF, OSM; Luminex, R&D Systems) or by enzyme-linked immunosorbent assay (IL-23, LegendMax, Biolegend). Each sample was measured in duplicate.

### Pathway analysis

Pathway analysis was performed using ingenuity pathway analysis (IPA; Qiagen Inc.; Krämer *et al.*, 2014). Given that our biomarkers are targeted for immune and neurological responsiveness, they specifically test a subset of immune and neurological pathways within IPA (Supplementary Table 3). To prevent subsequent bias, only those pathways in which 5 or greater of the 182 biomarkers were present in the Olink panel were analysed. Biomarkers were regarded as being upregulated if they had a fold change of 2 or greater and a significant *P-*value compared to the control group.

To simulate the effect of TLR activation during an inflammatory response, only the proteins identified within IPA as TLR responding were set to their levels in the infected response and this was overlaid onto the response in non-infected inflammatory patients. Changes were cascaded through the causal networks and pathway analysis was repeated with the updated protein levels. Pathways upregulated were then compared to the original pathway changes occurring in inflamed or infected patients to determine whether this was sufficient to recapitulate differences between the two patient groups.

### Statistical analysis

Statistical analyses and graphing were performed in Graphpad Prism 8.3.1, Morpheus (https://software.broadinstitute.org/morpheus/), and R version 3.6.2. Where multiple comparisons were performed, Benjamini–Hochberg correction with a false discovery rate of 5% was used unless otherwise stated.

To rank biomarkers for potential utility, the mean and average variation (MAV) score

$$\mathrm{MAV}=\left|{\overset{-}{x}}_{A}-{\overset{-}{x}}_{B}\right|-\left|\frac{n_{A}\sigma_{A}+\;n_{B}\sigma_{B}}{n_{A+B}}\right|$$

was used, where all readings are expressed as log2, and the two groups for comparison are A and B.

### Data availability

Data are available from the corresponding author on request.

## Results

### Patients

Between March 2017 and March 2018, 38 patients provided informed consent for use of their clinically sampled serum and CSF for research purposes. As categorized, 24 (63%) were ‘control’ patients, 19 with idiopathic intracranial hypertension and 5 with normal pressure hydrocephalus. 15/24 (62.5%) of these patients were shunted naïve (10 idiopathic intracranial hypertension and 5 normal pressure hydrocephalus) and 9/24 (37.5%) patients had either lumbo-peritoneal or VP shunts, none of which were suspected of infection (Table 1).

In the index group of 14 patients, 8 (21%) were ‘inflamed’ patients (4 with chemical meningitis post-cranial surgery and 4 with subarachnoid haemorrhage) with no evidence of infection and sterile CSF culture, and 6 (16%) were categorized as ‘infected’ patients (Table 1). The infected group included two patients whose CSF showed Gram-positive cocci and in whom coagulase-negative *Staphylococcus* spp. infection was confirmed by extended culture. Four patients did not have culture-positive CSF but clinical symptoms, serum and CSF markers (polymorphonuclear leukocytosis) consistent with meningitis/ventriculitis (Tunkel *et al.*, 2017) and were therefore treated on clinical grounds (Supplementary Table 1). They included one patient following trans-sphenoidal resection of a pituitary adenoma who developed a CSF leak and subsequent meningitis, one patient with an EVD *in situ* for hydrocephalus secondary to a colloid cyst, and a third patient with meningitis during an admission for subarachnoid haemorrhage. All three patients were treated with antibiotics for infection (two lower respiratory tracts and one urinary tract) prior to developing clinical symptoms and signs of meningitis. The fourth patient presented with a clinically infected pseudomeningocele following a posterior fossa tumour resection and had already commenced antibiotics. CSF was taken on insertion of a lumbar drain and was turbid in appearance with 86% polymorphonuclear leucocytosis on microscopy.

CSF white cell counts were raised significantly in inflamed patients and raised further in infected patients (Table 1). However, there was significant overlap between the groups and hence white cell counts alone was not sufficiently distinct for diagnostic purposes to discriminate between an inflamed or infective cause.

### IL-6 related biomarkers are elevated in both inflammation and infection and are correlated to CSF white cell numbers.

**Figure 1:**
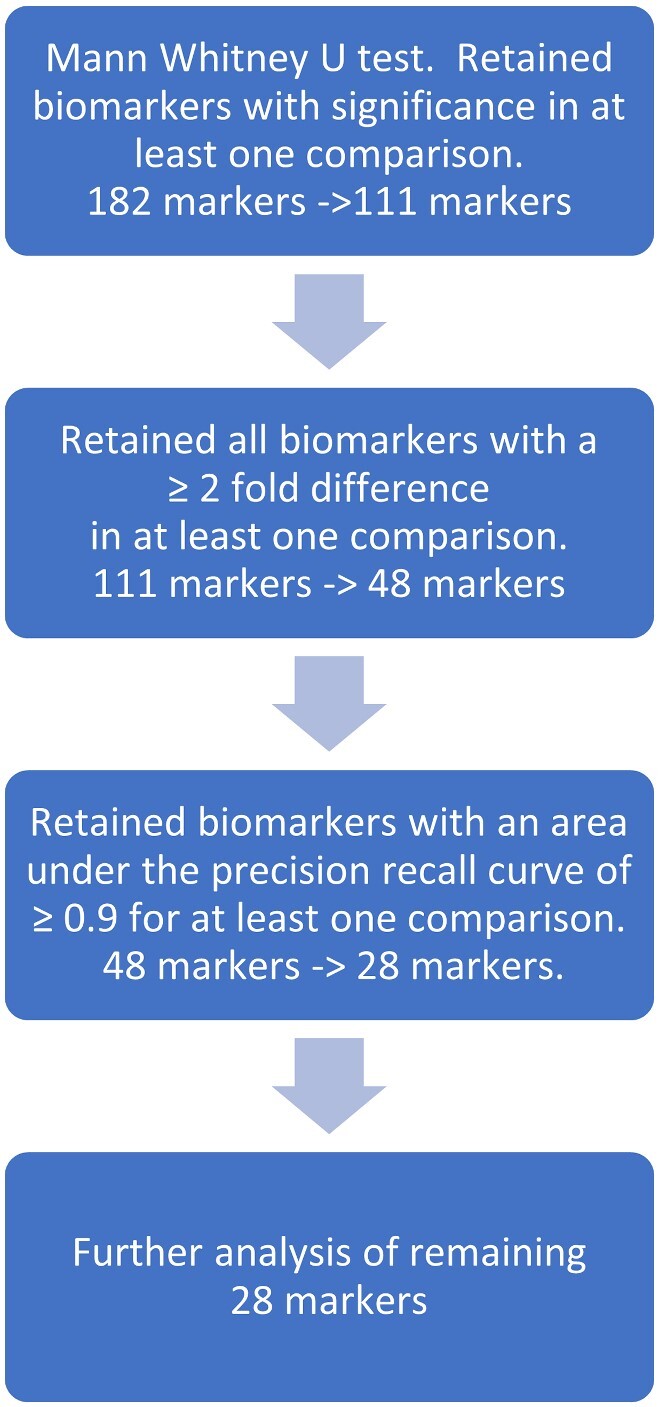
Plan of feature selection.

**Figure 2:**
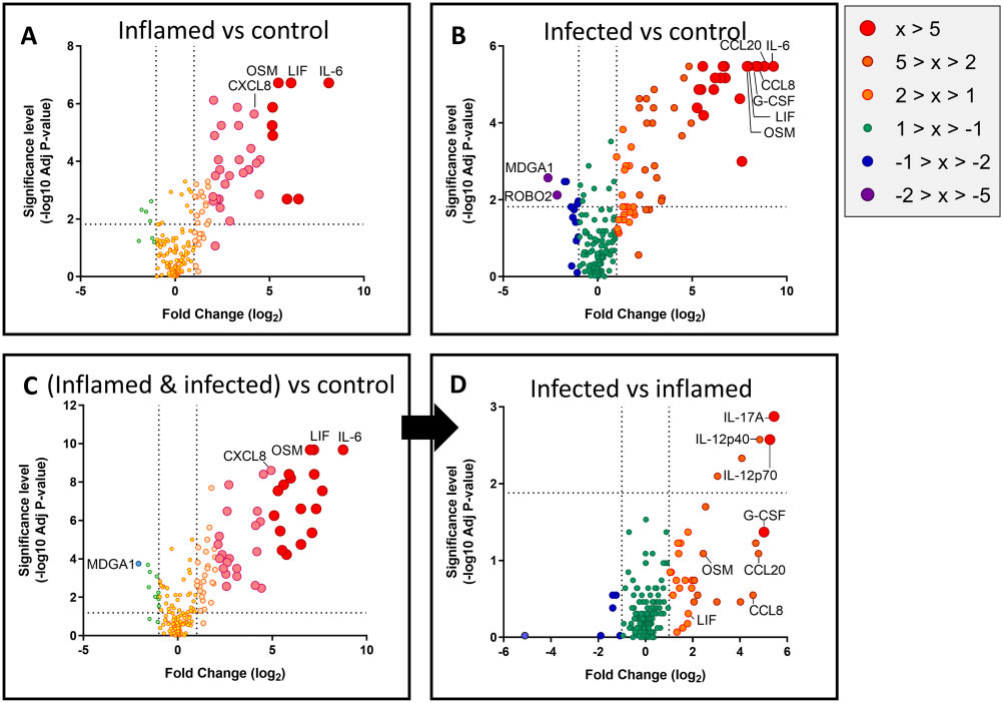
**Differential levels of proteins in the CSF.** Each protein assayed is indicated by an individual data point, with larger fold change in protein levels indicated by colour and size. Those with particularly large fold changes are most likely to be significant and are labelled. Horizontal line represents the Benjamini–Hochberg critical value at which statistical significance is achieved.

In order to examine differences in soluble proteins present in the CSF, we measured 182 neurological and immunological biomarkers (Supplementary Table 2) in all three patient groups. Given the relatively small number of patients, it was particularly important to reduce the dimensionality of the data for analysis while retaining those biomarkers with the potential for effective discrimination between groups. The schema used is shown in Fig. 1. Initially, biomarker levels were compared between groups for fold change and significant difference (Fig. 2). Those which were significant by Mann–Whitney test and with ≥ 2-fold change in expression between any two groups were retained, reducing the markers to be taken forward for further analysis to 48 candidates. As a next step, we used precision-recall curves which give similar information to a more conventionally used receiver operating characteristic curve but unlike receiver operating characteristic curves are not biased by large numbers of correctly classified negative control samples. This was an important consideration given that there were more patients in the control group than within other individual groups. Retaining only those biomarkers that showed an area under the precision-recall curves of 0.9 and above, the number of potentially relevant biomarkers was reduced further to a total of 28 proteins.

**Figure 3:**
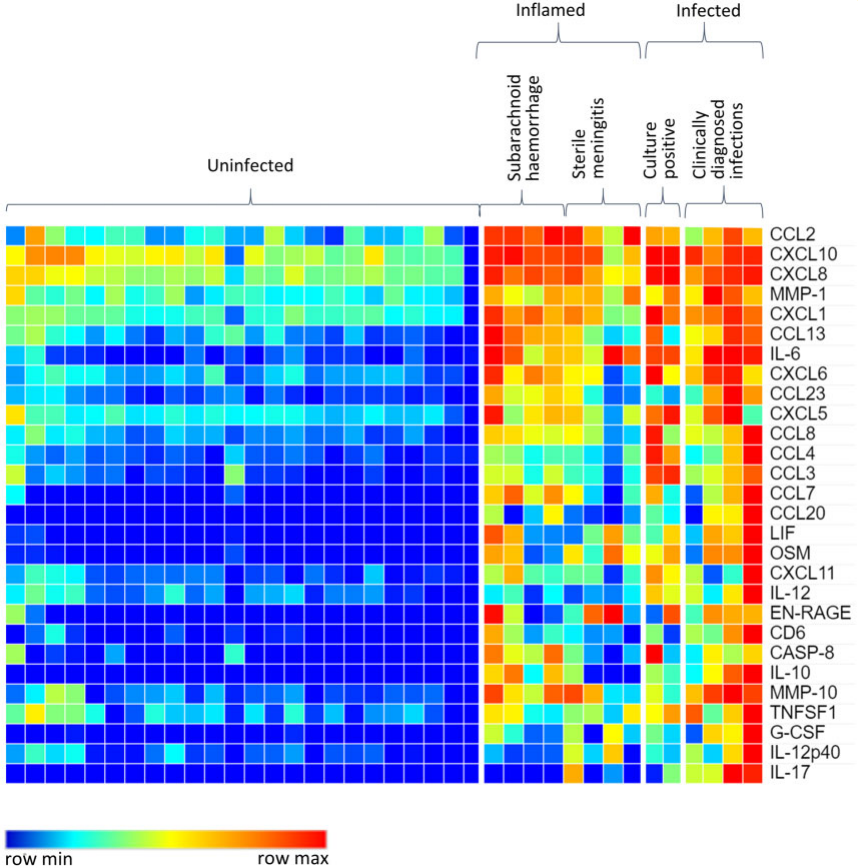
**Protein levels of biomarkers in the CSF.** Heat map showing relative amounts of immunological biomarkers in response to inflammation or infection compared to control patients. Higher concentrations of immunological biomarkers are indicated in red; lower concentrations are indicated in increasing blue intensity.

Overall, the biomarkers that were increased in inflamed and infected patients were very similar, with differences mainly occurring in the relative amount of each biomarker present rather than their identity (Fig. 2A and B). To find differences of potential diagnostic relevance between inflamed and infected patients despite this substantial overlap, a two-stage process was used in which inflamed and infected samples were combined and treated as one group compared to controls, and then the infected and inflamed groups were compared to one another as a second step (Fig. 2C and D). This two-stage process resulted in 28 markers showing differences between control and all infected/inflamed patients as described above (Fig. 3, Table 2). The most clearly differentiating biomarker between control patient samples and either bacterial infection or non-bacterial inflammation was IL-6. While the IL-6 related cytokines leukaemia inhibitory factor (LIF) and oncostatin M (OSM) appeared to have similar fold increases in concentration when looking at the aggregate data (Fig. 2), these were more variable when individual patient data were examined (Fig. 3). However, for all three biomarkers, levels were indistinguishable between infected and inflamed patients.

**Figure 4:**
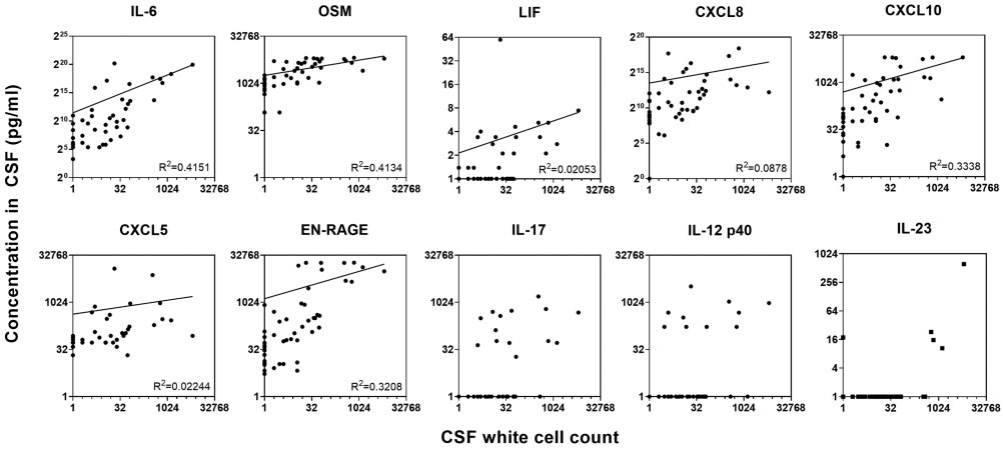
**Correlation between white cell count in the CSF and soluble biomarkers.** Cytokines shown were quantified by enzyme-linked immunosorbent assay (IL-23) or luminex (all other) and analysed using log-log regressions.

Given that CSF inflammation was associated with both an increase in inflammatory cytokines and increased leukocyte counts, we hypothesized that the two would be correlated and quantitated the most relevant proteins using the Luminex platform. Indeed, the amounts of the IL-6 related proteins were strongly correlated to cell numbers (Fig. 4). Similarly, the neutrophil-derived biomarker extracellular newly identified receptor for advanced glycation end-products binding protein (EN-RAGE/S100A12) had a clear association with infiltrating cells, reflecting the large number of neutrophils attracted into the sites after damage (*R*^2^ = 0.3208). The chemokine CXCL10 had a monotonic relationship with cell number (*R*^2^ = 0.3338), which was present but weaker for CXCL8 (*R*^2^ = 0.0878), consistent with their roles in attracting immune cells into sites of damage and inflammation.

### A distinct biomarker signature in cerebrospinal fluid identifies bacterial infection

Importantly, this two-stage process resulted in a subset of four markers showing differences between bacterially infected and non-specifically inflamed patients (Figs 2 and 3). Comparison of the inflamed and infected patients showed that CSF levels of IL-17, IL-12p40 and IL-12p70 successfully differentiated the two groups, with CCL3 showing lesser difference (Table 2). Interestingly, CCL20, CCL8 and granulocyte-colony stimulating factor, which were amongst the most differentiating biomarkers between controls and infected patients (Fig. 2B), were not statistically different between infected and inflamed patients, as shown by their relatively high *P*-values and correspondingly low −log10 *P*-values (Fig. 2D).

In contrast to IL-6 and related cytokines, IL-12p40 and IL-17 levels were not associated with infiltrating cell numbers, supporting a different aetiology for their production independently of the number of immune cells entering the site of inflammation (Fig. 4). Given that the IL-12p40 subunit is common to the dimeric cytokines IL-12p70 and IL-23, and the importance of the IL-17/IL-23 axis in neuroimmunology, we also quantitated IL-23 in patient samples. Similar to IL-12p40 and IL-17, IL-23 levels were not correlated with the number of immune cells in the CSF (Fig. 4).

Of note, measurement of serial samples in two control patients, one patient during and after chemical meningitis, and two patients during and after bacterial infection showed that none of these biomarkers changed with time, reinforcing the hypothesis that these markers were specific to infection and inflammation and not simply artefacts of variability between patients (Supplementary Fig. 1).

### IL-17 pathways are stimulated in all inflammation, but IL-17 levels are raised specifically during bacterial infection in response to TLR activation

**Figure 5:**
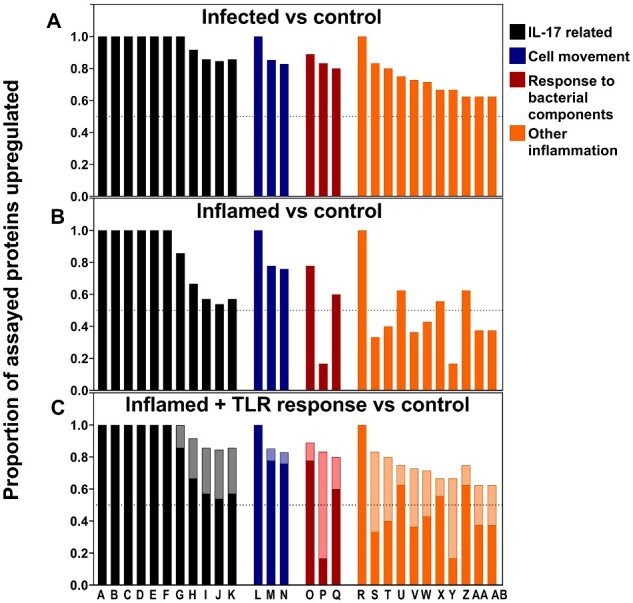
**Pathways potentially stimulated in CNS inflammation and infection.** (**A**) Pathways upregulated in infected patients compared to control patients. (**B**) Pathways upregulated in patients with CNS inflammation compared to control patients. (**C**) The resultant pattern of pathway stimulation if the proteins increased in the TLR pathway are added into the biomarkers responding in inflamed patients. Inflamed response shown in darker colours superimposed on the response to inflammation with the response to TLR stimulation added in paler colours. Labels A to AB on the x-axis refer to the pathways shown in Table 3.

To examine the underlying biology of the responses, pathway analyses were performed on the data. Changes in protein levels were compared between infected versus control groups, inflamed versus control groups and infected versus inflamed groups for all biomarkers with a significant *P-*value and greater than 2-fold change. Perhaps unsurprisingly given the sharing of many biomarkers between infection-related and non-infective inflammation (Supplementary Fig. 2), the identities of the pathways induced was broadly similar. Biomarkers found to be at increased concentrations in inflamed and infected patients were overwhelmingly found to contribute to IL-17-associated pathways, IL-6-associated inflammation and cell movement (Fig. 5A and B). All pathways upregulated were dominated by chemokines (Supplementary Table 3).

The most significant difference between inflamed and infected patients was in the induction of the TLR signalling pathway (Fig. 5B, column P) in which five of six proteins assayed were induced in infected patients compared to only one in inflamed patients. Given that many of the pathways shared common biomarkers, it was possible that induction of TLR signalling was artificially causing other pathways to appear to be induced in infected patients. To test this, the six biomarkers identified by the IPA pathway analysis program as being responsive to TLR stimulation were set to the higher levels seen in infected patients. These were then overlaid onto the response to inflammation in inflamed patients to simulate TLR activation in an inflamed environment, and the pathway analysis was repeated. The simulated TLR response was then compared to the response in infected patients. These calculations demonstrated unambiguously that adding the biomarkers associated with a TLR response to the biomarkers associated with the underlying inflammation in the inflamed patients recapitulated the pattern of pathway stimulation seen in the infected group (Fig. 5C). Overall, these data strongly support the response to the infection being characterized by an inflammatory response, which is augmented by TLR activation.

Upstream causal network analysis showed peptidoglycan to be the most likely upstream regulator in bacterially infected patients (*P *=* *5.87 × 10^−36^). Peptidoglycan is a component of the outer wall of bacteria which can be recognized by pattern recognition receptors such as TLR2 (Kielian *et al.*, 2005; Wolf and Underhill, 2018). This contrasted with the upstream regulators in inflammation which were overwhelmingly cytokines: IL-17F (*P *=* *1.05 × 10^−30^), IL-1α (*P *=* *4.23 × 10^−30^) and IL-4 (*P *=* *1.68 × 10^−29^). Together, the pathway analyses support TLR activation by peptidoglycan as the dominant pathway leading to increased inflammatory markers in infection compared to non-infectious inflammation.

### An efficient biomarker signature can be created from immune molecules present in the CSF

**Figure 6:**
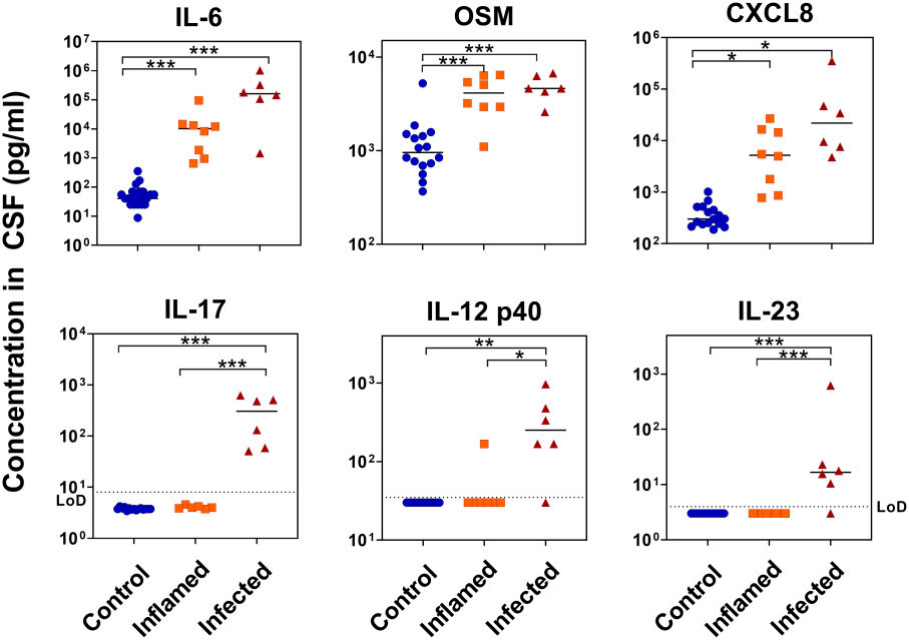
**Quantitation of biomarkers.** Biomarkers were measured in CSF by Luminex or enzyme-linked immunosorbent assay, and results were compared by Kruskall–Wallis followed by Dunn’s multiple comparisons test. * *P *<* *0.05, ** *P *<* *0.01, *** *P *<* *0.001.

To define a potential signature for diagnostic discrimination between non-bacterial inflammation and bacterial infection, biomarkers were ranked by comparison of their difference between means with averaged variation in subgroups (mean and average variation score). This was performed both for the discrimination between controls versus all inflamed and infected patients pooled together, and for the specific discrimination between inflamed versus infected patients. For validation of the raised levels in the Olink proximity extension assay, the absolute levels of the three proteins yielding the highest scores in each category were quantitated by Luminex or enzyme-linked immunosorbent assay and compared between groups as candidate signature molecules for discriminating inflammation and infection. Interestingly, while LIF had a receiver operating characteristic area under the curve of 1 absolute levels of LIF were very small, resulting in a low mean and average variation score that reflected its impracticality as a biomarker. In accordance with the relative biomarker levels determined by Olink analysis, absolute amounts of IL-6, OSM and CXCL8 were effective indicators of inflammation without discriminating between bacterial and non-bacterial causes of inflammation (Fig. 6). IL-6 was capable of distinguishing patients with inflammation (bacterial or non-bacterial) from control patients with a sensitivity and specificity of 100%; OSM with a sensitivity of 92.9% and specificity of 93.8%; and CXCL8 with a sensitivity of 85.7% and specificity of 100%. In contrast, elevated levels of IL-17, IL-23 and IL-12p40 were capable of predicting infection specifically, with IL-17 (100% sensitivity and specificity) more effective than IL-23 (83.3% sensitivity, 100% specificity) and IL-12p40 (83.3% sensitivity, 85.7% specificity) for this cohort. Taken together, these findings support a potential biomarker signature of diagnostic relevance, consisting of raised IL-6, OSM and CXCL8 in the CSF for detecting general CNS inflammation, and additional raised IL-17 as well as IL-12p40 or IL-23 as indicative of a bacterial cause for the inflammation. As the functional IL-23 cytokine contains the IL-12p40 subunit, we speculate that for practical reasons it will be sufficient, and arguably more specific, in future studies to measure directly IL-23 instead of IL-12p40.

## Discussion

In this study, we investigated a panel of 182 immune and neurological markers using advanced IPA pathway analysis and identified a biomarker signature distinguishing nosocomial infection from neuroinflammation, based on the biologically plausible pathway of TLR activation by bacterial cell wall products. If validated in a larger clinical study, this signature could be of significant value for allowing rapid point of care diagnosis of infections, facilitating the difficult task of determining whether a patient will benefit from antibiotics or require a different therapy. This study also gives an intriguing insight into early pathways differentially activated by sterile inflammation and nosocomial infection, both converging on a common downstream inflammatory response which may explain many of the previous difficulties in distinguishing these important conditions and points to the potential utility of biomarker strategies targeting early differential initiating pathways.

While limited patient numbers mean that our study does not have sufficient statistical power to definitively describe the biomarkers and their specificities and sensitivities, our pathway analyses strongly support that the responding biomolecules represent real differences in the underlying biology between the groups rather than false-positive readings. Further, the inclusion of a wide range of mixed pathologies supports that the responding biomolecules represent real differences and commonalities in the underlying biology between the diverse groups, making diversity a strength of the study. The value of the study is both in its examination of a wide battery of biomarkers to aid pathway analysis and in its selection of CSF samples taken at the time of clinical equipoise for the early diagnosis of nosocomial infection from a wide background of clinical pathologies. CSF analysis is unavoidably skewed by the clinical decision to sample, and the equipoise examined can remain after the initial CSF cell counts are available while definitive microbiological culture is awaited. This is indicative of the real-world clinical difficulty, unlike that examined in many studies which seek to diagnose nosocomial infection either purely on the basis of CSF examination, the sampling timing of which is not clear relative to the diagnostic equipoise (Beer *et al.*, 2008; Muñoz-Gómez *et al.*, 2015; Sorinola *et al.*, 2019; Zheng *et al.*, 2020) or later purely on a limited number of biomarkers analysed *posthoc* (van Mourik *et al.*, 2012, 2015). The multiple pathologies we encountered are also reflective of clinical practice where the ultimate goal is to achieve diagnostic resolution of nosocomial infection against the complete spectrum of non-infective aetiologies encountered in neurosurgical and neurological care.

As an accurate diagnosis of nosocomial infection in all cases is so challenging (Dorresteijn *et al.*, 2019), we, like others, chose a pragmatic approach of either bacteria seen on Gram stain or positive bacteriological culture or a highly suggestive clinically picture with supportive CSF pleocytosis and a response to antibiotic treatment. In our study, the utility of examining additional CSF biomarkers at the point of equipoise after initial CSF analysis is the pertinent question and the accuracy or otherwise of the initial clinical impression is secondary to the primary comparison of the eventual outcomes with the CSF biomarker pattern and pathway analysis.

Elevated CSF levels of IL-6 have been well described in studies of community-acquired bacterial meningitis (Prasad *et al.*, 2014; García-Hernández *et al.*, 2016; Srinivasan *et al.*, 2016; Lepennetier *et al.*, 2019; Liba *et al.*, 2019), but not in all (Asano *et al.*, 2010), and IL-6 is also raised in encephalitis (Morichi *et al.*, 2018). Elevated IL-6 levels in CSF have not only been reported in nosocomial infections in patients with shunts and EVDs (Asi-Bautista *et al.*, 1997; Schoch *et al.*, 2008; Lenski *et al.*, 2019, Skar *et al.*, 2019*b*) but also in inflammation and vasospasm after subarachnoid haemorrhage (Lenski *et al.*, 2017), all combining to make it a poor differentiator between nosocomial infection and sterile neuroinflammation. EN-RAGE is produced by macrophages in response to IL-6 *in vitro* (Hasegawa *et al.*, 2003), and both EN-RAGE and IL-6 (Chiaretti *et al.*, 2008) have previously been noted as increased in serum or CSF, respectively, in response to cerebral trauma and during paediatric CNS infection when compared to controls (Prasad *et al.*, 2014; García-Hernández *et al.*, 2016). Given that production of IL-6 is a broad response to both trauma and immunological challenge, it is not surprising that it is elevated irrespective of whether CNS damage is sterile or induced by a bacterial or viral infection. This is all consistent with our findings of elevated IL-6 levels in both inflamed and infected groups and our pathway analysis identifying downstream IL-6 elevation in both groups.

Elevated CSF IL-17 has been reported in bacteriological CNS infections (Asano *et al.*, 2010; Prasad *et al.*, 2014), and in influenza-associated encephalopathy and convulsions following gastroenteritis (Morichi *et al.*, 2018). A single study has reported a non-significant trend to increased IL-17 CSF levels in nosocomial shunt related CSF infections (Skar *et al.*, 2019*b*). Here we show that IL-17 is particularly promising for differentiating infection from sterile inflammation, and the addition of variables such as IL-23 and IL-12p40 may further improve this differentiation. It is worth noting that generic IL-17 pathways were upregulated in both inflammation and infection despite IL-17 levels near or even below the enzyme-linked immunosorbent assay /Luminex detection limit in inflamed patients, in contrast to the higher levels found in bacterially infected patients. The upregulation of the generic IL-17 pathways in inflamed patients is instead indicated by increases in the chemokines that accompany IL-17 in the pathway. In contrast, increased IL-17 as an indicator of bacterial infection occurred in addition to the biomarkers increased in the canonical TLR pathways used in this study (see Supplementary Table 3). The source of IL-17 may include Th17 cells although four out of six IL-17 high patients in the present study had predominantly neutrophil responses in the CSF, which occur typically prior to optimal T cell responses. The pattern of accompanying cytokines and chemokines in fact suggests that IL-17 may alternatively derive from activated astroglial cells (Waisman *et al.*, 2015) or resident γδ T cells (Ribeiro *et al.*, 2019).

The finding that IL-23 is upregulated in the same patients as IL-17 is consistent with an overall stimulation of the IL-23/IL-17 axis, which has previously been found to be activated by peptidoglycan (Tyler *et al.*, 2017). Mouse studies have identified IL-23 as an important regulator in inducing IL-17 (Shichita *et al.*, 2009; Sutton *et al.*, 2009; Gelderblom *et al.*, 2018) and some forms of CNS inflammation (Cua *et al.*, 2003; Sutton *et al.*, 2009; Gelderblom *et al.*, 2018) in experimental systems. In patients, elevated IL-23 in CSF has been found in paediatric meningitis patients (Srinivasan *et al.*, 2018). While the authors of that study did not measure IL-17 or compare with sterile inflammation, the data are supportive of the hypothesis that the IL-17/IL-23 axis is broadly upregulated in patients with meningitis, as is a large hospital study into meningitis (albeit without comparing to unrelated inflammation) (Liu *et al.*, 2020). Our data form an important adjunct to the preclinical literature, showing that these findings are also likely to be relevant during human disease and linking studies which have examined the cytokines.

Our findings define an innate immune response to damage in the CSF of all patients with inflammation, which is overlaid with a TLR-stimulated response in the case of bacterial infection. The TLR response that was evident in bacterially infected patients is consistent with the reported expression of TLRs on microglia, oligodendrocytes and astrocytes (Bsibsi *et al.*, 2002; Jack *et al.*, 2005). TLRs are a family of evolutionarily ancient pattern recognition receptors which can recognize damage-associated molecular patterns and pathogen-associated molecular patterns, including lipoproteins and peptidoglycan from the outer surface of Gram-positive organisms such as *Staphylococcus* spp. Coagulase-negative staphylococci were identified in both patients in whom bacterial culture was successful, and are known to be a common cause of medical implant and shunt infection (Becker *et al.*, 2014; Jamjoom *et al.*, 2018; Mallucci *et al.*, 2019). The pathway analysis used in IPA indeed showed that TLRs are likely to be important in the induction of the cytokines and chemokines observed. Given the potential of peptidoglycan (Tyler *et al.*, 2017) and *S. aureus* extracts (Zielinski *et al.*, 2012) in upregulating IL-23 expression in monocytes and DCs and priming T cell responses, there is a potential pathway of action occurring in these patients that merits further biological validation.

In terms of defining potential biomarkers, previous studies identified CXCL8 and tumour necrosis factor-α as potentially useful in determining whether patients have bacterial CNS infection (Mukai *et al*., 2006; Hsieh *et al*., 2009; Pinto Junior *et al.*, 2011; García-Hernández *et al.*, 2016; Srinivasan *et al.*, 2016, 2018), although none of these were sufficiently predictive for differentiating infection from inflammation. While a comprehensive review is outside the scope of this study, it is worth highlighting a complementary approach to ours of examining inflammatory markers at the terminal pathways for bacteriological attack, such as the complement system’s soluble membrane attack complex (sMAC) (Ramos *et al.*, 2016). Although that study showed that sMAC levels above a 43 ng/ml had a sensitivity and specificity of 93% and 86%, respectively, with area under the curve 0.966 for diagnosing pyogenic infections in children, it was much poorer at identifying low-grade commensal pathogens such as *Staphylococcus* spp. or *Propionibacterium acnes* infection which are a more frequent cause of nosocomial infections in adults with shunts, and the examined clinical pathologies were confined to infection. Another promising approach is to use markers of neutrophils, macrophages and mature monocytes as they infiltrate the brain during infection by examining soluble triggering receptor expressed on myeloid cells (TREM)-1 in the CSF (Gordon *et al.*, 2014), which showed a 100% sensitivity and 98% specificity for diagnosing EVD bacterial ventriculitis. However, their study did not attempt to distinguish infective from non-infective inflammation, and standard CSF glucose levels performed as well as sTREM-1.

## Conclusion

In contrast to previous studies, we examined a wide battery of inflammatory and neurological markers allowing pathway analysis across a spectrum of challenging pathologies which generate sterile neuroinflammation against which nosocomial infection is extremely challenging to diagnose. Within our data, the most reliable signature for non-bacterial inflammation was high levels of IL-6 with low levels of IL-17, and the most reliable signature for bacterial infection was high levels of IL-6 with high levels of IL-17. Notably, IL-6 could not distinguish between bacterial and non-bacterial inflammation. Pathway analysis provided a biologically plausible explanation of differential upstream initiation in infection by TLR signalling via IL-17 in addition to common IL-6 associated inflammation in sterile neuroinflammation. This is the first study in patients to demonstrate biomarkers distinguishing infection from background neuroinflammation, and to use accompanying data to investigate which pathways are responsible. The data are especially relevant to the commonly encountered pathologies such as subarachnoid haemorrhage and post-cranial neurosurgery for diagnosing secondary nosocomial infection. Given variability within populations, the IL-6 and IL-17 markers are more likely to be robust if combined into a metric also including OSM and IL-23. If replicated in a larger study these findings could find immediate clinical utility for the bedside diagnosis of infected CSF with significant improvements in patient care and outcomes. Point-of-care lateral flow devices, such as the one recently developed for diagnosing peritonitis (Goodlad *et al.*, 2020), would permit bedside diagnosis within minutes leading to a potential step change in the care of neurological infection.

## Supplementary material

Supplementary material is available at *Brain Communications* online.

## Supplementary Material

fcaa218_Supplementary_Data

## Abbreviations

AUC =: area under the curve
CCL =: chemokine (C-C motif) ligand
CXCL =: C-X-C motif chemokine
EN-RAGE =: extracellular newly identified receptor for advanced glycation end-products binding protein (S100A12)
EVD =: external ventricular drain
G-CSF =: granulocyte-colony stimulating factor
IIH =: idiopathic intracranial hypertension
IL =: interleukin
IPA =: ingenuity pathway analysis
LD =: lumbar drain
LIF =: leukaemia inhibitory factor
LP =: lumbar puncture
MAV =: mean and average variation
NPH =: normal pressure hydrocephalus
OSM =: oncostatin M
SAH =: subarachnoid haemorrhage
TLR =: toll-like receptor
VP =: ventriculoperitoneal
WCC =: white cell counts
