# Supplementary material for: Toll-like receptor linked cytokine profiles in cerebrospinal fluid discriminate neurological infection from sterile inflammation
Source: Brain Commun. 2020 Dec 17;2(2):fcaa218. doi: 10.1093/braincomms/fcaa218 (PMC7772097; doi:10.1093/braincomms/fcaa218)
Supplement: fcaa218_Supplementary_Data [file fcaa218_supplementary_data.zip › Supplementary_tables.pdf]

Supplementary Tables

Groups		Neurological Diagnosis	Indication for CSF sample	Method of sampling
**CONTROLS N=24**	Shunt naïve	10 IIH<br>5 NPH	Symptom control	9 LP + 1 VP shunt<br>2 VP shunt + 3 LD
	Shunt in-situ	9 IIH	Shunt malfunction **	5 LP + 1 shunt tap + 3 shunt revision
**INFLAMED N=8**	Vascular	4 SAH	Hydrocephalus	1 EVD + 3 LP
	Post-surgical	1 Trigeminal neuralgia<br>1 Epidermoid cyst<br>2 Chiari malformation	Chemical Meningitis	All LP
**INFECTED N=6**	Culture positive	1 Haemangioblastoma<br>1 IIH	Meningitis<br>Infected shunt	1 LD<br>Shunt revision
	Culture negative	1 Pituitary adenoma<br>1 Haemangioblastoma<br>1 Colloid cyst<br>1 SAH	Meningitis<br>Infected Pseudomeningocele<br>Meningitis/Ventriculitis<br>Meningitis	LP<br>LD<br>EVD<br>LP

Supplementary Table 1: Indications for sampling according to groupings and method of sampling.
** 6 patients went on to have shunt revision.
IIH – idiopathic intracranial hypertension
NPH – Normal pressure hydrocephalus
SAH – subarachnoid haemorrhage
LP – lumbar puncture
VP – ventriculoperitoneal
LD – lumbar drain
EVD – External Ventricular Drain

Supplementary Table 2. Complete list of soluble biomarkers screened

4E-BP1	CD244	EZR	IL-12	NCAN	SLAMF1
α2-MRAP	CD355	FcRL2	IL-12p40	N-CDase	SMOC2
ADA	CDCP1	FGF-5	IL-13	NEP	SMPD1
ADAM 22	CDH3	FGF-19	IL-15RA	NMNAT1	SPOCK1
ADAM 23	CDH6	FGF-21	IL-17	Nr-CAM	ST1A1
ARTN	CLEC1B	FGF-23	IL-17C	NRP2	STAMBP
AXIN1	CLEC10A	FLRT2	IL-18	NRTN	TGF-α
BCAN	CLM-1	Flt3L	IL-18R1	NT-3	TGF-β1 proprotein
BMP-4	CLM-6	gal-8	IL-20	NTRK2	THY 1
β-NGF	CNTN5	GCP5	IL-20RA	NTRK3	TMPRSS5
CADM3	CPA2	G-CSF	IL-22RA1	OPG	TNFRSF12A
CASP-8	CPM	GDF-8	IL-24	OSM	TNFRSF21
CCL2	CSF-1	GDNF	IL-33	PDGF-R-α	TNFRSF27
CCL3	CST5	GDNFRα3	JAM-B	PD-L1	TNFRSF9
CCL4	CTSC	GFR-α1	KYNU	PLXNB1	TNFSF1
CCL7	CTSS	GM-CSF-Rα	LAIR-2	PLXNB3	TNFSF10
CCL8	CX3CL1	GZMA	LAT	PRTG	TNFSF11
CCL11	CXCL1	HAGH	LAYN	PVR	TNFSF12
CCL13	CXCL5	HGF	LIF	RGMA	TNFSF14
CCL19	CXCL6	IFNγ	LIF-R	RGMB	TNFα
CCL20	CXCL8	IL-1α	LXN	ROBO2	TN-R
CCL23	CXCL9	IL-2	MANF	RSPO1	TSLP
CCL25	CXCL10	IL-2RB	MAPT	SCARA5	UNC5C
CCL28	CXCL11	IL-4	MATN3	SCARB2	uPA
CD5	DDR1	IL-5	MDGA1	SCARF2	VEGFA
CD6	Dkk-4	IL-5Rα	MMP-1	SCF	VWC2
CD8A	DNER	IL-6	MMP-10	sFRP-3	WFIKKN1
CD38	DRAXIN	IL-7	MSR1	Siglec-1	
CD40	EFNA4	IL-10	N2DL-2	Siglec-9	
CD200	EN-RAGE	IL-10RA	NAAA	SIRT2	
CD200R1	EPHB6	IL-10RB	NBL1	SKR3	

Supplementary Table 3. Canonical pathways with 5 or biomarkers present in the analysis.

**Ingenuity Canonical Pathways**	**Molecules**
Actin Cytoskeleton Signalling	EZR, FGF19, FGF21, FGF23, FGF5
Activation of IRF by Cytosolic Pattern Recognition Receptors	CD40, IL10, IL6, LTA, TNF
Acute Phase Response Signalling	IL18, IL1A, IL33, IL6, OSM, TNF, TNFRSF11B
Adrenomedullin signalling pathway	IL18, IL1A, IL33, LTA, TNF
Agranulocyte Adhesion and Diapedesis	CCL11, CCL13, CCL19, CCL2, CCL20, CCL23, CCL25, CCL28, CCL3, CCL4, CCL7, CCL8, CX3CL1, CXCL1, CXCL10, CXCL11, CXCL5, CXCL6, CXCL8, CXCL9, EZR, IL18, IL1A, IL33, MMP1, MMP10, TNF
Allograft Rejection Signalling	CD40, IFNG, IL10, IL2, IL4, IL5, TNF
Altered T Cell and B Cell Signalling in Rheumatoid Arthritis	CD40, CSF1, IFNG, IL10, IL12A, IL12B, IL17A, IL18, IL1A, IL2, IL33, IL4, IL6, LTA, SLAMF1, TGFB1, TNF, TNFSF11
Atherosclerosis Signalling	CCL11, CCL2, CD40, CSF1, CXCL8, IFNG, IL18, IL1A, IL33, IL6, MMP1, MSR1, TGFB1, TNF, TNFRSF12A, TNFSF12, TNFSF14
Autoimmune Thyroid Disease Signalling	CD40, IL10, IL2, IL4, IL5
Axonal Guidance Signalling	BMP4, EFNA4, EPHB6, MMP1, MMP10, NGF, NRP2, NTRK2, NTRK3, PLXNB1, PLXNB3, ROBO2, UNC5C, VEGFA
Bladder Cancer Signalling	CXCL8, FGF19, FGF21, FGF23, FGF5, MMP1, MMP10, VEGFA
Cardiac Hypertrophy Signalling (Enhanced)	CXCL8, FGF19, FGF21, FGF23, FGF5, IFNG, IL10RA, IL10RB, IL12A, IL12B, IL13, IL15RA, IL17A, IL17C, IL18, IL18R1, IL1A, IL2, IL20RA, IL22RA1, IL2RB, IL33, IL4, IL5, IL5RA, IL6, LIF, LTA, OSM, TGFB1, TNF, TNFRSF11B, TNFSF10, TNFSF11, TNFSF12, TNFSF14
Chemokine Signalling	CCL11, CCL13, CCL2, CCL4, CCL7
Clathrin-mediated Endocytosis Signalling	FGF19, FGF21, FGF23, FGF5, VEGFA
Colorectal Cancer Metastasis Signalling	AXIN1, IFNG, IL6, MMP1, MMP10, TGFB1, TNF, VEGFA
Communication between Innate and Adaptive Immune Cells	CCL3, CCL4, CD40, CD8A, CXCL10, CXCL8, IFNG, IL10, IL12A, IL12B, IL18, IL1A, IL2, IL33, IL4, IL5, IL6, TNF
Crosstalk between Dendritic Cells and Natural Killer Cells	CD40, IFNG, IL12A, IL12B, IL15RA, IL18, IL2, IL2RB, IL4, IL6, LTA, TNF, TNFSF10
Death Receptor Signalling	CASP8, TNF, TNFRSF21, TNFSF10, TNFSF12
Dendritic Cell Maturation	CD40, IL10, IL18, IL1A, IL12A, IL12B, IL33, IL6, LTA, TNF, TNFRSF11B
Differential Regulation of Cytokine Production in Intestinal Epithelial Cells by IL-17A and IL-17F	CCL2, CCL3, CCL4, CSF3, CXCL1, IFNG, IL10, IL12A, IL12B, IL13, IL17A, IL1A, TNF

Differential Regulation of Cytokine Production in Macrophages and T Helper Cells by IL-17A and IL-17F	CCL2, CCL3, CCL4, CSF3, CXCL1, IL10, IL12A, IL12B, IL13, IL17A, IL6, TNF
FAT10 Cancer Signalling Pathway	IFNG, IL6, TGFB1, TNF, TNFRSF11B
Fc Epsilon RI Signalling	IL13, IL4, IL5, LAT, TNF
FGF Signalling	FGF19, FGF21, FGF23, FGF5, HGF
FXR/RXR Activation	FGF19, IL18, IL1A, IL33, TNF
Glucocorticoid Receptor Signalling	CCL11, CCL13, CCL2, CCL3, CXCL8, IFNG, IL10, IL13, IL2, IL4, IL5, IL6, MMP1, TGFB1, TNF
Graft-versus-Host Disease Signalling	IFNG, IL18, IL1A, IL2, IL33, IL6, TNF
Granulocyte Adhesion and Diapedesis	CCL11, CCL13, CCL19, CCL2, CCL20, CCL23, CCL25, CCL28, CCL3, CCL4, CCL7, CCL8, CSF3, CX3CL1, CXCL1, CXCL10, CXCL11, CXCL5, CXCL6, CXCL8, CXCL9, EZR, IL18, IL1A, IL33, MMP1, MMP10, TNF, TNFRSF11B
Hematopoiesis from Multipotent Stem Cells	CSF1, CSF3, IL2, IL4, IL5, IL7
Hematopoiesis from Pluripotent Stem Cells	CD8A, CSF1, CSF3, CXCL8, IL10, IL1A, IL2, IL4, IL5, IL6, IL7, IL12A, IL12B, LIF
Hepatic Cholestasis	CXCL8, FGF19, IFNG, IL13, IL17A, IL17C, IL18, IL1A, IL2, IL33, IL4, IL5, IL6, IL12A, IL12B, LIF, LTA, OSM, TGFB1, TNF, TNFRSF11B, TNFSF10, TNFSF11, TNFSF12, TNFSF14
Hepatic Fibrosis / Hepatic Stellate Cell Activation	CCL2, CD40, CSF1, CXCL8, CXCL9, HGF, IFNG, IL10, IL10RA, IL1A, IL4, IL6, MMP1, PDGFRA, TGFA, TGFB1, TNF, TNFRSF11B, VEGFA
HMGB1 Signalling	CCL2, CXCL8, IFNG, IL12A, IL12B, IL13, IL17A, IL17C, IL18, IL1A, IL2, IL33, IL4, IL5, IL6, LIF, LTA, OSM, TGFB1, TNF, TNFRSF11B, TNFSF10, TNFSF11, TNFSF12, TNFSF14
Human Embryonic Stem Cell Pluripotency	AXIN1, BMP4, NGF, NTRK2, NTRK3, PDGFRA, TGFB1
IL-10 Signalling	IL10, IL10RA, IL10RB, IL18, IL1A, IL33, IL6, TNF
IL-12 Signalling and Production in Macrophages	CD40, IFNG, IL10, IL12A, IL12B, IL18, IL4, TGFB1, TNF
IL-15 Production	DDR1, IL6, NTRK2, NTRK3, PDGFRA
IL-15 Signalling	CXCL8, IL15RA, IL17A, IL2RB, IL4, IL6, TNF
IL-17 Signalling	CCL11, CCL2, CXCL1, CXCL10, CXCL11, CXCL5, CXCL8, IL17A, IL6
IL-17A Signalling in Airway Cells	CCL11, CCL20, CXCL1, CXCL5, CXCL6, IL17A, IL6
IL-17A Signalling in Fibroblasts	CCL2, CCL7, CXCL5, IL17A, IL6, MMP1
IL-17A Signalling in Gastric Cells	CCL20, CXCL1, CXCL10, CXCL11, CXCL8, IL17A, TNF
IL-23 Signalling Pathway	IL12B, IL17A, TNF, TNFSF11
IL-6 Signalling	CXCL8, IL18, IL1A, IL33, IL6, TNF, TNFRSF11B, VEGFA

LPS/IL-1 Mediated Inhibition of RXR Function	IL18, IL1A, IL33, TNF, TNFRSF11B
LXR/RXR Activation	CCL2, CCL7, IL18, IL1A, IL33, IL6, MSR1, TNF, TNFRSF11B
MSP-RON Signalling Pathway	CCL2, CSF1, IFNG, TNF, IL12A, IL12B
Neuroinflammation Signalling Pathway	CASP8, CCL2, CCL3, CD200, CD200R1, CD40, CX3CL1, CXCL10, CXCL8, GDNF, IFNG, IL10, IL12A, IL12B, IL18, IL4, IL6, MAPT, NGF, NT3, TGFB1, TNF
NF-κB Signalling	BMP4, CASP8, CD40, IL18, IL1A, IL33, LTA, NGF, NTRK2, NTRK3, PDGFRA, TGFA, TNF, TNFRSF11B, TNFSF11
Osteoarthritis Pathway	CASP8, CXCL8, MATN3, MMP1, MMP10, TGFB1, TNF, VEGFA
p38 MAPK Signalling	IL18, IL1A, IL33, MAPT, TGFB1, TNF
Pathogenesis of Multiple Sclerosis	CCL3, CCL4, CXCL10, CXCL11, CXCL9
PD-1, PD-L1 cancer immunotherapy pathway	IFNG, IL2, IL2RB, IL4, IL12A, IL12B, LAT, TGFB1, TNF, TNFRSF11B
PPAR Signalling	IL18, IL1A, IL33, PDGFRA, TNF, TNFRSF11B
Regulation of the Epithelial-Mesenchymal Transition Pathway	AXIN1, FGF19, FGF21, FGF23, FGF5, HGF, TGFB1
Role of Cytokines in Mediating Communication between Immune Cells	CSF3, CXCL8, IFNG, IL10, IL13, IL17A, IL18, IL1A, IL12A, IL12B, IL2, IL20, IL24, IL33, IL4, IL5, IL6, TGFB1, TNF
Role of Hypercytokinemia/ Hyperchemokinemia in the Pathogenesis of Influenza	CCL2, CCL3, CCL4, CXCL10, CXCL8, IFNG, IL12A, IL12B, IL17A, IL18, IL1A, IL33, IL6, TNF
Role of IL-17A in Arthritis	CCL2, CCL20, CCL7, CXCL1, CXCL5, CXCL6, CXCL8, IL17A, MMP1
Role of IL-17A in Psoriasis	CCL20, CXCL1, CXCL5, CXCL6, CXCL8, IL17A
Role of IL-17F in Allergic Inflammatory Airway Diseases	CCL2, CCL4, CCL7, CXCL1, CXCL10, CXCL5, CXCL6, CXCL8, IL6
Role of JAK1 and JAK3 in γc Cytokine Signalling	IL15RA, IL2, IL2RB, IL4, IL7, TSLP
Role of Macrophages, Fibroblasts and Endothelial Cells in Rheumatoid Arthritis	AXIN1, CCL2, CSF1, CXCL8, DKK4, IL10, IL17A, IL18, IL18R1, IL1A, IL33, IL6, IL7, LTA, MMP1, OSM, TGFB1, TNF, TNFRSF11B, TNFSF11, VEGFA
Role of Osteoblasts, Osteoclasts and Chondrocytes in Rheumatoid Arthritis	AXIN1, BMP4, CSF1, DKK4, IFNG, IL10, IL17A, IL18, IL18R1, IL1A, IL33, IL4, IL6, IL7, MMP1, TGFB1, TNF, TNFRSF11B, TNFSF11
Role of Pattern Recognition Receptors in Recognition of Bacteria and Viruses	CXCL8, IFNG, IL10, IL12A, IL12B, IL13, IL17A, IL17C, IL18, IL1A, IL2, IL33, IL4, IL5, IL6, LIF, LTA, OSM, TGFB1, TNF, TNFSF10, TNFSF11, TNFSF12, TNFSF14
Role of Tissue Factor in Cancer	CSF1, CXCL1, CXCL8, FGF5, MMP1, VEGFA

STAT3 Pathway	HGF, IL10RA, IL10RB, IL15RA, IL18R1, IL1A, IL20RA, IL22RA1, IL2RB, IL5RA, NTRK2, NTRK3, PDGFRA, TGFA, TGFB1, VEGFA
Synaptogenesis Signalling Pathway	CDH3, CDH6, EFNA4, EPHB6, MAPT, NTRK2
Systemic Lupus Erythematosus in B Cell Signalling Pathway	CD40, CD5, CXCL8, IFNG, IL10, IL12A, IL12B, IL13, IL17A, IL17C, IL18, IL1A, IL2, IL33, IL4, IL5, IL6, LIF, LTA, OSM, TGFB1, TNF, TNFSF10, TNFSF11, TNFSF12, TNFSF14
Systemic Lupus Erythematosus in T Cell Signalling Pathway	CASP8, EZR, IL10, IL12B, IL17A, IL2, IL6, LAT
Systemic Lupus Erythematosus Signalling	CD40, IL10, IL18, IL1A, IL2, IL33, IL6, LAT, TNF
T Cell Exhaustion Signalling Pathway	IFNG, IL10, IL10RA, IL10RB, IL12A, IL12B, IL6, TGFB1, VEGFA
T Helper Cell Differentiation	CD40, IFNG, IL10, IL10RA, IL10RB, IL12A, IL12B, IL13, IL17A, IL18, IL18R1, IL2, IL4, IL5, IL6, TGFB1, TNF, TNFRSF11B
Tec Kinase Signalling	TNF, TNFRSF21, TNFSF10, TNFSF12
Th1 and Th2 Activation Pathway	CD40, CD8A, IFNG, IL10, IL10RA, IL10RB, IL12A, IL12B, IL13, IL18, IL18R1, IL2, IL24, IL2RB, IL33, IL4, IL5, IL6, LTA, TGFB1, TNFSF11, TSLP
Th1 Pathway	CD40, CD8A, IFNG, IL10, IL10RA, IL10RB, IL12A, IL12B, IL18, IL18R1, IL2, IL4, IL6, LTA, TNFSF11
Th17 Activation Pathway	CCL20, IFNG, IL10, IL12A, IL12B, IL17A, IL6
Th2 Pathway	CD40, IFNG, IL10, IL12A, IL12B, IL13, IL2, IL24, IL2RB, IL33, IL4, IL5, TGFB1, TSLP
Thyroid Cancer Signalling	CXCL8, GDNF, NGF, NTRK2, NTRK3
Toll-like Receptor Signalling	IL12A, IL12B, IL18, IL1A, IL33, TNF
TREM1 Signalling	CCL2, CCL3, CCL7, CD40, CXCL8, IL10, IL18, IL6, TNF
Type I Diabetes Mellitus Signalling	CASP8, IFNG, IL2, LTA, TNF, TNFRSF11B
VDR/RXR Activation	CXCL10, IFNG, IL2, IL12A, TNFSF11
