# Supplementary material for: Toll-like receptor linked cytokine profiles in cerebrospinal fluid discriminate neurological infection from sterile inflammation
Source: Brain Commun. 2020 Dec 17;2(2):fcaa218. doi: 10.1093/braincomms/fcaa218 (PMC7772097; doi:10.1093/braincomms/fcaa218)
Supplement: fcaa218_Supplementary_Data [file fcaa218_supplementary_data.zip › Supplementary_figures.pdf]

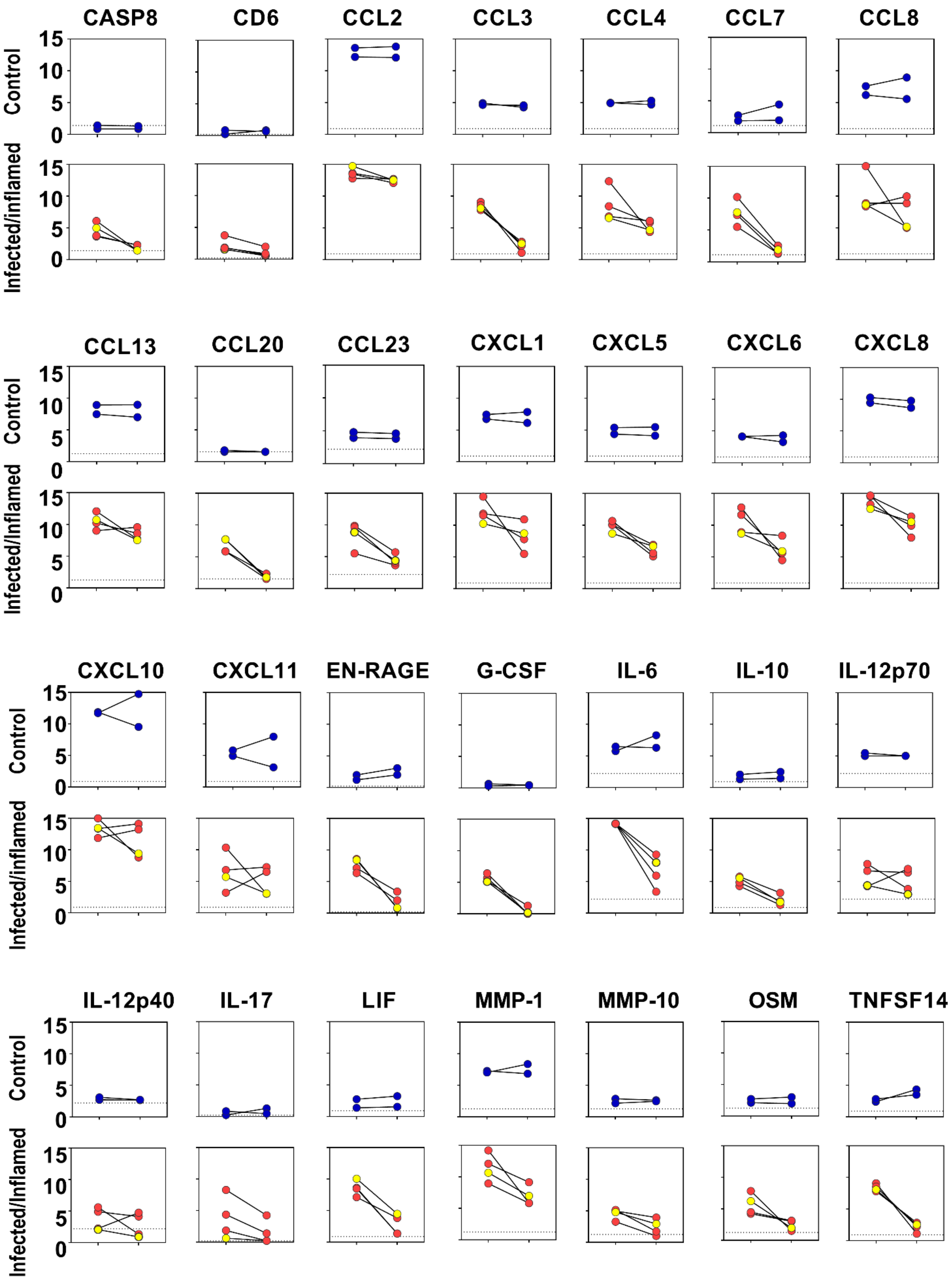

Supplementary Figure 1. Comparison of intra-patient vs inter-patient differences in CSF biomarker levels. Serial samples were obtained from control patients (blue), one patient during and after chemical meningitis (yellow), and from two patients during and after bacterial infection (red). For infected and inflamed patients, samples taken at timepoint 1 were taken during the inflammatory episode and those at timepoint 2 were taken after recovery. Y axis represents NPX units.

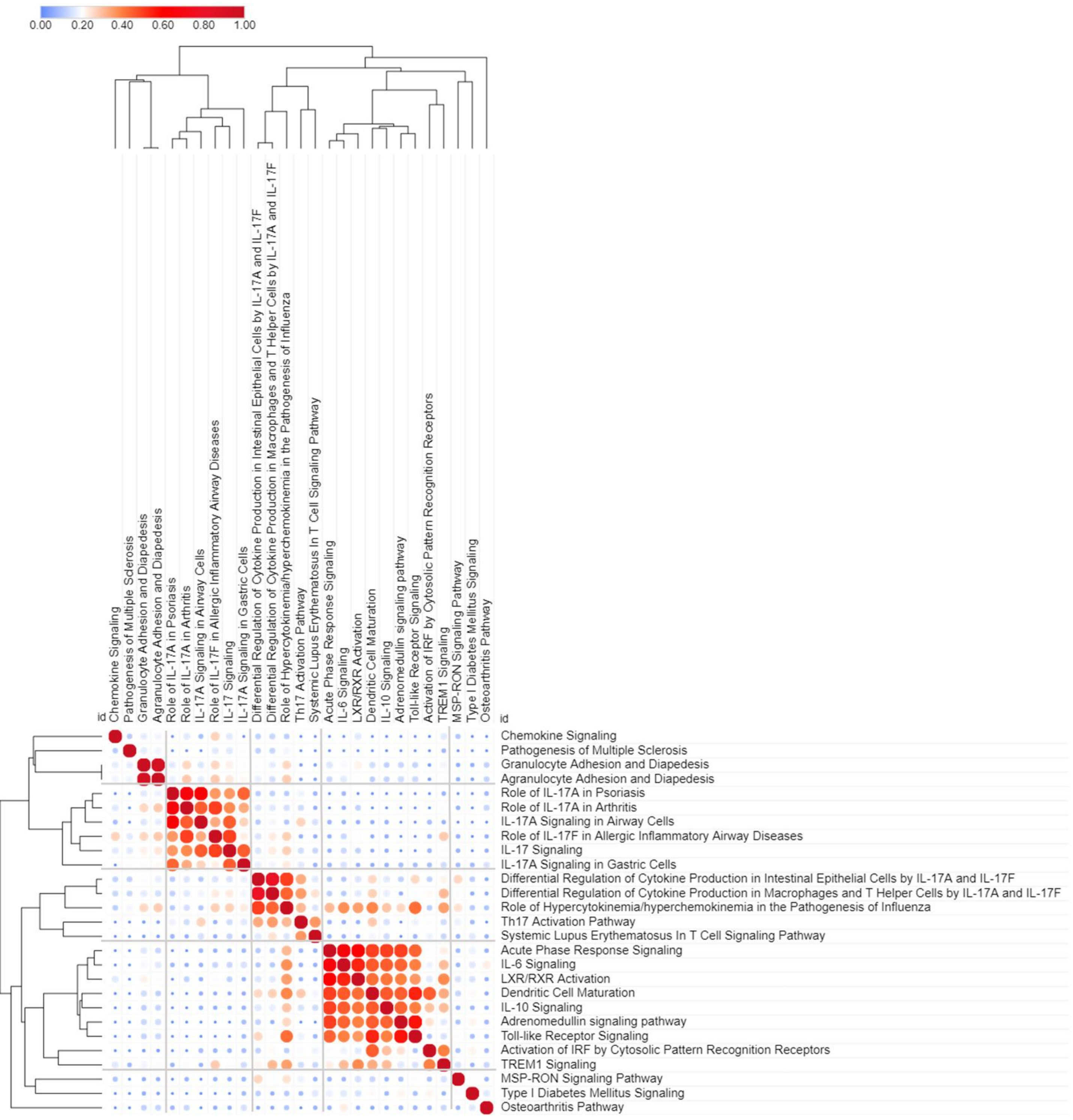

Supplementary Figure 2. Similarity matrix of pathways found to be upregulated. Matrix is based on the sharing of contributory proteins measured in this study.
